# Marked elevation of adrenal steroids, especially androgens, in saliva of prepubertal autistic children

**DOI:** 10.1007/s00787-013-0472-0

**Published:** 2013-09-17

**Authors:** Maria Dorota Majewska, Martin Hill, Ewa Urbanowicz, Paulina Rok-Bujko, Przemysław Bieńkowski, Irena Namysłowska, Paweł Mierzejewski

**Affiliations:** 1 Department of Pharmacology and Physiology of the Nervous System, Institute of Psychiatry and Neurology, Sobieskiego 9, 02-957 Warsaw, Poland; 2 Institute of Endocrinology, Prague, Czech Republic; 3 Department of Child and Adolescent Psychiatry, Institute of Psychiatry and Neurology, Warsaw, Poland

**Keywords:** Autism, Children, Saliva, Steroids, Neurosteroids

## Abstract

Autism is diagnosed on the basis of behavioral manifestations, but its biomarkers are not well defined. A strong gender bias typifying autism (it is 4–5 times more prevalent in males) suggests involvement of steroid hormones in autism pathobiology. In order to evaluate the potential roles of such hormones in autism, we compared the salivary levels of 22 steroids in prepubertal autistic male and female children from two age groups (3–4 and 7–9 years old) with those in healthy controls. The steroids were analyzed using gas chromatography–mass spectrometry and radioimmunoassay. Statistical analysis (ANOVA) revealed that autistic children had significantly higher salivary concentrations of many steroid hormones (both C21 and C19) than control children. These anomalies were more prominent in older autistic children and in boys. The levels of androgens (androstenediol, dehydroepiandrosterone, androsterone and their polar conjugates) were especially increased, indicative of precocious adrenarche and predictive of early puberty. The concentrations of the steroid precursor, pregnenolone, and of several pregnanolones were also higher in autistic than in healthy children, but cortisol levels were not different. Some steroids, whose levels are raised in autism (allopregnanolone, androsterone, pregnenolone, dehydroepiandrosterone and their sulfate conjugates) are neuroactive and modulate GABA, glutamate, and opioid neurotransmission, affecting brain development and functioning. These steroids may contribute to autism pathobiology and symptoms such as elevated anxiety, sleep disturbances, sensory deficits, and stereotypies among others. We suggest that salivary levels of selected steroids may serve as biomarkers of autism pathology useful for monitoring the progress of therapy.

**Electronic supplementary material:**

The online version of this article (doi:10.1007/s00787-013-0472-0) contains supplementary material, which is available to authorized users.

## Introduction

Autism and autism spectrum disorders (ASDs) encompass a group of behaviorally defined neurodevelopmental disorders typified by impairments of communication, social withdrawal, emotional deficits, high anxiety, stereotyped behaviors, movement and sensory dysfunctions, which may or may not be accompanied by cognitive deficits, various neurological and psychiatric comorbidities, as well as neuroanatomical and clinical anomalies. The incidence of ASDs has been rising at an alarming rate over the past three decades, although the prevalence seems to differ between countries [1]. In the UK and several other European countries, current prevalence rates of ASDs approach 1 % [1, 2], while in the US, according to recent CDC estimates, the prevalence among children 6–17 years old in 2011–2012 was 2 % (and 3.2 % in boys; (http://www.cdc.gov/nchs/data/nhsr/nhsr065.pdf). Such data are not available for Poland, but reports from diagnostic and treatment clinics indicate that these rates are also rising. Yet, the etiology of autism is poorly understood and little is being done to prevent this devastating pediatric disorder. Multiple hypotheses on autism etiology—including genetic and epigenetic origin, prenatal or postnatal brain injuries or infections, immune and mitochondrial dysfunctions, in addition to environmental and iatrogenic causes—probably reflect the biological heterogeneity of ASDs as well as lack of in-depth understanding of autism pathogenesis [3–6].

Autism is about four to five times more common in males than in females [7, 8] and some investigators view autistic traits as manifestations of an “extreme male brain” [9]. Among many biological factors, which may lay at the basis of this gender bias, the genetic and hormonal influences seem most obvious and should be thoroughly examined to gain an understanding of autism pathobiology. Several studies showed anomalous steroid metabolism in autism, but the data are inconsistent: some documented androgen excess in autistic children and adults [10, 11], other showed androgen deficits [12, 13], or no difference between autistic and control children [14]; although the latter group found hyperandrogenism in autistic children with aggression [15]. Furthermore, some investigators reported a dysfunctional HPA axis in autistic persons, manifested by increased plasma levels of ACTH, but reduced levels of cortisol [16]; others found augmented cortisol secretion in autistic children in response to novel stimuli [17]. Aberrant metabolism of cholesterol [18] and altered expression of steroid metabolizing genes [19] were also described in autistic patients.

Notwithstanding certain inconsistencies between different studies which may reflect genetic/biological heterogeneity, diverse ages of studied cohorts, varied environmental and pharmaceutical exposures, or dissimilarities in diagnostics and other experimental conditions; most studies point to dysregulated steroid metabolism in autism. While the causes of these anomalies may be manifold, an abnormal steroid milieu is likely to play a role in the pathogenesis and clinical/behavioral manifestations of autism, because steroids participate in development of the brain and control many of its functions. Steroids exert organizational and activational actions during brain development [20, 21] and modulate neurotransmission either by directly interacting with neurotransmitter receptors or by genomic mechanisms [22–25].

In the present study, we compared the levels of an array of salivary steroids in prepubertal male and female autistic Polish children to those of their sex- and age-matched controls. We chose saliva as the specimen for investigation, because it can be collected non-invasively and non-stressfully; this is particularly important for autistic children, who are very prone to stress. The experimental cohorts included the same children as in the previously published study, in which we compared mercury levels in the hair of autistic and control children [26]. In analyzing the steroids we paid particular attention to androgens, glucocorticoids and neuroactive steroids (or neurosteroids), which regulate neurotransmission of GABA and glutamate [22, 23, 27, 28], among other neuronal actions. Our results show markedly increased levels of many steroids in the saliva of autistic children. We discuss potential roles of these hormones in pathogenesis and manifestations of clinical/behavioral autism symptoms and suggest that measurement of selected salivary steroids may be a promising, non-invasive biochemical test helpful in monitoring the progress of autism treatment.

## Methods

### Study design

The study was carried out in accordance with the Helsinki declaration of the Worlds Medical Association and the protocol was approved by the Ethics Committee of the Institute of Psychiatry and Neurology in Warsaw. Involvement in the study was voluntary and was not compensated. The parents of patients read and signed informed consent forms after the study procedures had been fully explained to them. Autistic and control male and female children from two age groups, 3–4 and 7–9 years old were recruited from the children earlier diagnosed with autism in out-patient clinics. Healthy control children were recruited from six preschools and three primary Warsaw schools. Enrollment took place from November 2007 to April 2009. All participants were Caucasians. Patients were excluded if they had a neurological and psychiatric disorder other than autism and comorbid disorders; history of liver, renal or endocrine disorder; current infection or fever. Mental retardation or behavioral disorders were exclusion criteria only for the control group, but were allowed as comorbid condition in the autistic cohort. Children diagnosed with Asperger’s syndrome were excluded. Patients were not tested for Fragile X or other genetic defects.

### Clinical evaluation

Each autistic child was rediagnosed by a team of two experienced psychologists and one child psychiatrist according to the Diagnostic and Statistical Manual of Mental Disorders (DSM–IV) criteria for autism or pervasive developmental delay. The activity and functioning of an autistic child were also assessed according to the Childhood Autism Rating Scale (CARS) [29] and the Clinical Global Impression Scale. Autistic children who scored 30 or more points in CARS were enrolled. Extensive medical histories of study participants were collected. Control participants were assessed with use of the Abbreviated Parent-Teacher Questionnaire to exclude children with symptoms of ADHD. Details of diagnostic procedures and data collection were described previously [26].

### Steroid analysis

The steroids were purchased from Steraloids (Newport, RI, USA), the Sylon B from Supelco (Bellefonte, PA, USA), the methoxylamine hydrochloride from Sigma (St. Louis, MO, USA) and the solvents from Merck (Darmstadt, Germany). Children’s saliva (0.15–5 mL) was collected at home by parents (to avoid stress), between the hours of 8:00 and 10:00 AM before breakfast into sterile plastic tubes using salivettes (Sarstedt). It was immediately frozen, and during the following 5 days delivered on ice to the laboratory, where it was thawed, filtered using sterile Millipore filters, and frozen again in Eppendorf tubes. It was kept in the freezer (at −70 °C) until shipment on dry ice to the Institute of Endocrinology in Prague for steroid analysis. The saliva samples from autistic and control children were collected concurrently during the study recruitment period. They were treated in exactly the same way and were shipped to Prague simultaneously. All steroid analyses were conducted blindly.

The preparation of samples for further processing was as follows: saliva was kept frozen at −20 °C until analysis. Samples were thawed, the 20 μL of solution of internal standard (17α-estradiol, 100 pg/μL) was added, centrifuged at 3,000–4,000 g for 10 min, and the clear supernatant was extracted with a triple volume of diethyl ether. The aqueous phase was frozen in a mixture of solid carbon dioxide and ethanol and the extracts were decanted into glass tubes and evaporated to dryness. To analyze the free steroids and the steroid polar conjugates, the dry residue as well as the supernatants were further processed and analyzed in the same way as described in our recently published method for steroid analysis in maternal and fetal serum and amniotic fluid [30] with some modifications. (1) The amount of internal standard added to the polar fraction after diethyl ether extraction was 20 μL of 17α-estradiol 100 pg/μL instead of 20 μL of 17α-estradiol 1 ng/μL, and the final volume of derivatized sample with fraction of steroid polar conjugates was 50 μL instead of 200 μL. (2) Some steroids like estrogens, progesterone, testosterone were not measured due to problems with sensitivity. (3) Androstenediol was measured together with 5α-androstane-3β, 17β-diol (gradient G3) and not with 5α/β-androstane-20-oxo steroids (androsterone, etiocholanolone, etc.—gradient G1).

Steroid analysis was performed using the GCMS-QP2010 Plus system from Shimadzu (Kyoto, Japan), which consisted of a gas chromatograph equipped with automatic flow control, AOC-20s autosampler and a single quadrupole detector. The experimental details, effective masses, retention times of chromatographic peaks, sequence number of injection for steroid groups and gradients that were used for quantification of individual steroids are described in the previous publication [30]. Cortisol was assayed using a commercial kit Spectra^®^ from Orion Diagnostica (Espoo, Finland). The analytical range for saliva was 0–100 nm/L, limit of detection = 0.8 nmol/L, recovery = 110–136 %, intra-assay coefficient of variation (CV) = 4.5 %, inter-assay CV = 5.5 %. The concentrations of steroid conjugates were calculated for MW of free steroids.

To test if there were differences in steroid concentrations between fresh samples of saliva and those twice frozen and thawed, 20 replicates of pooled fresh saliva samples and the same number of replicate samples processed as the experimental samples were analyzed. The results analyzed with the Mann–Whitney test revealed no statistically significant differences in concentrations of all steroids between these two groups of samples (*p* > 0.05), indicating steroid stability in our experimental protocol.

### Statistical analysis

The STATISTICA software package (StatSoft, Tulsa, OK, USA) was used to analyze all data. The Student’s *t* test was used when means of data from two groups were compared. Mann–Whitney *U* test was used for comparisons of nonparametric data, McNemar’s test with Chi-square statistics was applied for categorical variables (‘yes’ or ‘no’). Results with *p* level less than 0.05 were considered significant. Demographic and clinical data are presented as mean ± standard error of mean (SEM).

Salivary steroid concentrations are shown as mean (nM) ± SEM and as median values. Differences in steroid levels between different groups were assessed with the aid of three-way (Illness × Sex × Age Group) analysis of variance (ANOVA) with Illness (Autism vs. Control), Sex (males vs. females), and Age Group (3–5 vs. 7–9 years old) as independent variables. Post hoc analyses were conducted using the Newman–Keuls test. *p* values less than 0.05 were considered significant.

## Results

### Demographic and clinical data

The study participants included 78 autistic and 70 healthy control children divided into 8 experimental groups: AM I and AM II (autistic males 3–4 and 7–9 years old, respectively), AF I and AF II (autistic females 3–4 and 7–9 years-old), CM I and CM II (control males 3–4 and 7–9 years-old), CF I and CF II (control females 3–4 and 7–9 years-old). Demographic and birth-related data comparing the groups are shown in Table 1. Overall, the groups of younger autistic children did not differ significantly from controls in demographics and birth-linked parameters; minor significant differences in mean age and maternal age at birth of one group (AF I) seem trivial. Among the four groups of older children, three groups also did not significantly differ in these parameters. The only exception was the group of older autistic girls (AF II), which was a few months younger than other groups (*p* = 0.008) and more disadvantaged at birth, as its members were born at a slightly earlier gestational age (*p* = 0.04), hence weighed less at birth (*p* = 0.01) and received lower Apgar scores (*p* = 0.03).

**Table 1:** Demographic and birth-related data of study participants

Demographic data	Males		Females		*p* (*t*-student)
	Autistic	Control	Autistic	Control	
Age groups I (3–4 years)	AM I	CM I	AF I	CF I	
*N*	23	19	22	16	
Mean age	3.7 ± 0.1	3.5 ± 0.1	3.9 ± 0.2*	3.4 ± 0.1	0.04
Weight at birth (g)	3,487 ± 118	3,467 ± 123	3,241 ± 76	3,239 ± 124	
Head circumference at birth (cm)	34.2 ± 0.3	34.0 ± 0.4	33.7 ± 0.3	33.3 ± 0.4	
Gestational age at birth (weeks)	38.8 ± 0.3	39.3 ± 0.4	39.1 ± 0.3	39.1 ± 0.4	
Apgar score	9.4 ± 0.3	9.5 ± 0.2	9.5 ± 0.4	10 ± 0	
Mother’s age at birth	28.8 ± 0.6	28.4 ± 0.9	27.7 ± 0.9*	31.1 ± 0.9	0.01
Father’s age at birth	30.7 ± 0.8	31.5 ± 1.0	29.6 ± 1.1	33.3 ± 1.4	

Age groups II (7–9 years)	AM II	CM II	AF II	CF II	
*N*	20	17	13	18	
Mean age	8.2 ± 0.2	8.4 ± 0.2	7.7 ± 0.2*	8.4 ± 1.4	0.008
Weight at birth (g)	3,272 ± 207	3,466 ± 159	2,756 ± 227*	3,417 ± 119	0.01
Head circumference at birth (cm)	33.9 ± 0.8	34.1 ± 0.5	32.1 ± 0.8	33.7 ± 0.4	
Gestational age at birth (weeks)	38.2 ± 0.8	38.7 ± 0.4	37.2 ± 1.0*	39.4 ± 0.4	0.04
Apgar score	8.5 ± 0.6	9.5 ± 0.2	8.7 ± 0.4*	9.6 ± 0.2	0.03
Mother’s age at birth	27.6 ± 0.8	27.8 ± 1.0	28.2 ± 1.6	27.0 ± 0.9	
Father’s age at birth	29.9 ± 0.9	29.6 ± 0.8	30.4 ± 1.3	28.5 ± 0.8	

AM I and AM II (autistic males age group I and II, respectively), CM I and CM II (control males age group I and II), AF I and AF II (autistic females age group I and II), CF I and CF II (control females age group I and II)

* Statistically significant differences between autistic and control groups

The data in Table 2 compare the clinical features of autistic and control children. Significantly more autistic than control children experienced developmental regress and manifested abnormal development (e.g. hypotonia, delayed sitting, walking, fine motor skills, speech delay or lack of it, periods of irregular breathing, sleep apnea, poor sleep, nightly screams, tantrums, severe digestive problems, diarrhea) and hyperactivity. Within their age groups, male and female autistic children did not vary significantly in the number of DSM-IV criteria or CARS scores. Abnormal development noted in three control children was mostly related to delayed speech and walking.

**Table 2:** Comparison of clinical features of autistic and control children from both age- and sex- groups

Clinical features	Males		Females		*p* (Chi-square)
Age groups I (3–4 years)	AM I	CM I	AF I	CF I	
Abnormal development (%)	25.0	5.3	31.8*	0	0.01
Regress (%)	83.0*	0	81.8*	0	<0.001
Hyperactivity (%)	63.0*	0	63.6*	0	<0.001
CARS total scores	44.0 ± 1.4		45.7 ± 1.3		
DSM IV A	9.5 ± 0.3		9.6 ± 0.2		

Age groups II (7–9 years)	AM II	CM II	AF II	CF II	
Abnormal development (%)	55.0	11.8	69.2*	5.6	0.05
Regress (%)	55.0*	0	76.9*	0	<0.001
Hyperactivity (%)	40.0*	0	53.8*	0	<0.001
CARS total scores	39.3 ± 1.2		42.8 ± 1.7		
DSM IV A	10.2 ± 0.2		10.1 ± 0.3		

AM I and AM II (autistic males age group I and II, respectively), CM I and CM II (control males age group I and II), AF I and AF II (autistic females age group I and II), CF I and CF II (control females age group I and II)

* Statistically significant differences between autistic and control groups

### Salivary Steroids

Differences in the salivary levels of 22 steroids between all experimental groups were assessed with the aid of three-way ANOVA, including illness, sex, and age as independent variables. The data presented as mean (nM) concentrations (±SEM) and median concentrations are shown in Table 3 (males) and Table 4 (females). The results of ANOVA are described in table legend and those of post hoc analyses are shown in tables.

**Table 3:** Salivary steroid concentrations in autistic and control male children

Steroid	AM I (Autism) *N* = 23	CM I (Control) *N* = 19	p*,** Autism/control	AM II (Autism) (*N* = 20)	CM II (Control) (*N* = 17)	p*,** Autism/control
**C21 steroids**						
Pregnenolone	2.675 (0.321) *2.457*	2.061 (0.171) *2.143*		4.454 (0.682)** *3.593* ^##^	1.295 (0.133) *1.264*	<0.01
Pregnenolone-C	8.261 (0.936) *8.387*	6.171 (1.030) *4.752*		9.931 (1.993) *6.611*	5.854 (0.551) *5.994*	
20α-Dihydropregnenolone	0.819 (0.102) *0.863*	0.652 (0.090) *0.517*		1.196 (0.141)** *1.068* ^*#*^	0.363 (0.046) *0.312* ^##^	<0.01
20α-Dihydopregnenolone-C	3.825 (0.494) *3.465*	1.891 (0.333) *1.575*		5.199 (0.976)* *4.333*	2.060 (0.598) *1.526*	<0.05
P3α5α (Allopregnanolone)	0.112 (0.010) *0.111*	0.130 (0.011) *0.110*		0.177 (0.029)** *0.145*	0.030 (0.008) *0.016* ^*##*^	<0.01
P3α5α-C (Allopregnanolone-C)	0.616 (0.162) *0.266*	0.799 (0.191) *0.410*		0.669 (0.169) *0.572*	0.744 (0.174) *0.377*	
P3β5α-C (Isopregnanolone-C)	4.392 (1.512) *2.661*	2.544 (0.274) *2.509*		5.364 (0.799)* 4.445*	3.108 (0.705) *2.099*	<0.05
P3β5β-C (Epipregnanolone-C)	2.901 (0.301) *2.790*	4.765 (1.106) *3.251*		4.587 (0.983)** *3.429*	2.045 (0.463) *1.429* ^*##*^	<0.01
Cortisol	3.332 (1.024) *2.421*	2.178 (0.288) *2.071*		3.844 (0.730) *2.597*	3.631 (0.381) *3.514*	
**C19 steroids**						
DHEA (Dehydroepiandrosterone)	2.282 (0.315) *1.944*	1.424 (0.221) *1.076*		5.522 (1.934)** *3.361* ^##^	0.880 (0.106) *0.799*	<0.01
DHEA-C (Dehydroepiandrosterone-C)	69.66 (30.05)* *16.229*	7.702 (1.440) *6.840*	<0.05	560.05 (278.11)** *43.198* ^*##*^	39.06 (23.82) *9.702* ^*##*^	<0.01
DHEA-7o	2.648 (0.301) *1.910*	1.795 (0.269) *1.766*		3.331 (0.691)* *2.607*	1.345 (0.199) *0.935*	0.05
Androstenediol	1.468 (0.171) *1.321*	1.015 (0.129) *0.859*		1.746 (0.206)** *1.599*	0.637 (0.091) *0.598* ^*#*^	<0.01
Androstendione	0.346 (0.059) *0.247*	0.496 (0.282) *0.179*		0.851 (0.526) *0.346*	0.258 (0.082) *0.150*	
A3α5α (Androsterone)	0.086 (0.015) *0.061*	0.044 (0.007) *0.036*		0.069 (0.019) *0.063*	0.087 (0.016) *0.068*	
A3α5α-C (Androsterone-C)	6.972 (1.767)* *3.756*	3.057 (0.770) *1.984*	0.010	9.919 (1.509)** *9.669*	1.379 (0.222) *1.074*	<0.01
A3α5β (Etiocholanolone)	0.043 (0.008) *0.041*	0.068 (0.024) *0.032*		0.072 (0.014)** *0.061*	0.015 (0.003) *0.012*	<0.01
A3α5β-C (Etiocholanolone-C)	2.286 (0.401) *1.429*	1.857 (0.327) *1.348*		3.085 (0.961)** *1.935*	0.533 (0.148) *0.265*	<0.01
A3β5α (Epiandrosterone)	0.156 (0.019) *0.166*	0.105 (0.012) *0.112*		0.402 (0.223)** *0.182* ^*##*^	0.075 (0.011) *0.064*	<0.01
A3β5α-C (Epiandrosterone-C)	5.917 (1.364) *4.392*	3.758 (0.399) *3.637*		22.22 (8.214)** *7.969* ^#^	2.062 (0.763) *0.914*	<0.01
AT-7α (5-Androstene-3β,7α,17β-triol)	0.589 (0.076)* *0.484*	0.308 (0.043) *0.266*	<0.05	0.569 (0.062)** *0.553*	0.186 (0.029) *0.159*	<0.01
AT-7β (5-Androstene-3β,7β,17β-triol)	0.373 (0.044)* *0.361*	0.220 (0.030) *0.180*	<0.05	0.410 (0.047)** *0.379*	0.168 (0.022) *0.138*	<0.01

Mean steroid concentrations (nM) ± (SEM). Italics—median concentrations (nM)

Analysis: three-way ANOVA—significant effects and interactions of Pregnenolone: illness [*F*(1,140) = 25.02, *p* < 0.01], sex [*F*(1,140) = 11.40, *p* < 0.01], and age group × illness interaction [*F*(1,140) = 20.91, *p* < 0.01], other effects were not significant (*p* > 0.05). Pregnenolone-C: illness [*F*(1,138) = 20.62, *p* < 0.01], sex [*F*(1,138) = 18.33, *p* < 0.01], age group × sex interaction [*F*(1,138) = 6.48, *p* = 0.01]. 20-alpha dihydropregnenolone: illness [*F*(1,133) = 33.63, *p* < 0.01], sex [*F*(1,133) = 69.78, *p* < 0.01], age group × illness interaction [*F*(1,133) = 11.68, *p* < 0.01]. 20-alpha dihydropregnenolone-C: illness [*F*(1,113) = 21.00, *p* < 0.01]. Allopregnanolone: illness [*F*(1,131) = 46.08, *p* < 0.01], sex [*F*(1,131) = 63.47, *p* < 0.01], age group [*F*(1,131) = 18.36, *p* < 0.01], age group × sex interaction [*F*(1,131) = 5.29, *p* = 0.02], age group × illness interaction [*F*(1,131) = 33.30, *p* < 0.01], age group × sex × illness triple interaction [*F*(1,131) = 14.38, *p* < 0.01]. Isopregnanolone-C: sex × illness interaction [*F*(1,104) = 4.4, *p* ≤ 0.04], age group × sex × illness triple interaction [*F*(1,104) = 5.1, *p* = 0.03]. Epipregnanolone-C: sex [*F*(1,139) = 100.8, *p* < 0.01], age group × sex × illness triple interaction [*F*(1,139) = 12.8, *p* < 0.01]. DHEA: illness [*F*(1,140) = 43.33, *p* < 0.01] and age group × illness interaction [*F*(1,140) = 14.78, *p* < 0.01]. DHEA-C: illness [*F*(1,139) = 36.74, *p* < 0.01], sex [*F*(1,139) = 10.43, *p* < 0.01], age group [*F*(1,139) = 21.43, *p* < 0.01]. DHEA-7o: illness [*F*(1,114) = 7.67, *p* < 0.01] and sex × illness interaction [*F*(1,114) = 5.04, *p* = 0.03]. Androstenediol: illness [*F*(1,140) = 45.37, *p* < 0.01], sex [*F*(1,140) = 4.23, *p* = 0.04], age group × illness interaction [*F*(1,140) = 7.46, *p* < 0.01]. Androsterone-C: sex [*F*(1,139) = 7.15, *p* < 0.01] and age group × illness interaction [*F*(1,139) = 4.89, *p* = 0.03]. Etiocholanolone: illness [*F*(1,111) = 5.4, *p* = 0.02] and age group × illness interaction [*F*(1,111) = 8.4, *p* < 0.01]. Etiocholanolone-C: illness [*F*(1,138) = 5.3, *p* = 0.02]. Epiandrosterone: illness [*F*(1,113) = 467.29, *p* < 0.01], sex [*F*(1,113) = 8.01, *p* < 0.01], sex × illness interaction [*F*(1,113) = 7.22, *p* < 0.01], age group × illness interaction [*F*(1,113) = 9.36, *p* < 0.01]. Epiandrosterone-C: illness [*F*(1,139) = 35.41, *p* < 0.01], sex [*F*(1,139) = 25.42, *p* < 0.01], age group [*F*(1,139) = 35.41, *p* < 0.01], age group × sex interaction [*F*(1,139) = 6.33, *p* = 0.01], age group × illness interaction [*F*(1,139) = 8.76, *p* < 0.01], age group × sex × illness triple interaction [*F*(1,139) = 6.87, *p* < 0.01]. 5-Androstene-3β,7α,17β-triol (AT-7alpha): illness [*F*(1,139) = 32.78, *p* < 0.01] and sex × illness interaction [*F*(1,139) = 5.6, *p* = 0.02]. 5-Androstene-3β,7β,17β-triol (AT-7beta): illness [*F*(1,140) = 26,25, *p* < 0.01]. Cortisol, allopregnanolone-C, androstenedione and androsterone: no significant effects

AMI and AM II (autistic males group I and II, respectively), CM I and CM II (control males group I and II)

* *p* < 0.05; ** p < 0.01, significant differences in steroid concentrations between autistic and control children within their age groups

^#^ *p* < 0.05; ^##^ *p* < 0.01 significant differences between older and younger children, either autistic or control, “C” after steroid name denotes conjugate

**Table 4:** Salivary concentrations of steroids in autistic and control female children

Steroid	AF I *N* = 22	CF I *N* = 16	p*, ** Autism/control	AF II *N* = 13	CF II *N* = 18	p*, ** Autism/control
**C21 steroids**						
Pregnenolone	1.783 (0.156) *1.561*	1.992 (0.315) *1.642*		2.256 (0.508)** *1.657*	1.160 (0.137) *1.216* ^#^	<0.01
Pregnenolone-C	8.483 (1.606) *5.401*	4.629 (0.988) *3.535*		6.301 (2.062)** *3.634*	2.529 (0.323) *2.112* ^*#*^	<0.01
20α-Dihydropregnenolone	0.614 (0.058) *0.533*	0.447 (0.051) *0.426*		0.679 (0.122)* *0.583*	0.339 (0.036) *0.349*	<0.05
20α-Dihydopregnenolone-C	3.496 (0.421) *3.365*	2.153 (0.487) *1.943*		3.366 (1.132) *1.698*	1.555 (0.239) *1.598*	
P3α5α (Allopregnanolone)	0.056 (0.010) *0.048*	0.028 (0.003) *0.026*		0.057 (0.020)** *0.039*	0.018 (0.002) *0.016*	<0.01
P3α5α-C (Allopregnanolone-C)	0.685 (0.150) *0.391*	0.889 (0.538) *0.284*		0.286 (0.072) *0.199*	0.378 (0.069) *0.304*	
P3β5α-C (Isopregnanolone-C)	3,077 (0.557) *2.045*	2.525 (0.901) *1.364*		1.402 (0.127) *1.592*	3.858 (1.764)* *2.407*	
P3β5β-C (Epipregnanolone-C)	0.848 (0.167) *0.636*	1.185 (0.456) *0.509*		0.474 (0.087) *0.413*	1.246 (0.312) *0.762*	
Cortisol	6.377 (3.508) *2.898*	2.736 (0.498) *2.337*		4.345 (1.042) *3.740*	3.963 (0.508) *3.128*	
**C19 steroids**						
DHEA (Dehydroepiandrosterone)	1.046 (0.112) *0.896*	0.916 (0.135) *0.803*		4.993 (3.267)* *1.330* ^*#*^	0.812 (0.104) *0.898*	<0.05
DHEA-C (Dehydroepiandrosterone-C)	24.404 (9.368)* *9.234*	4.363 (0.987) *3.417*	<0.05	575.70 (555.1) *14.562*	25.301 (9.567) *7.481* ^*#*^	
DHEA-7o	1.453 (0.195) *1.257*	1.600 (0.254) *1.706*		2.098 (0.650) *1.555*	1.687 (0.244) *1.853*	
Androstenediol	1.085 (0.091) *1.065*	0.745 (0.083) *0.612*		1.356 (0.379)** *1.042*	0.608 (0.059) *0.483*	<0.01
Androstendione	0.259 (0.044) *0.205*	0.373 (0.126) *0.166*		0.253 (0.087) *0.155*	0.166 (0.039) *0.116*	
A3α5α (Androsterone)	0.074 0.014) *0.049*	0.063 (0.014) *0.043*		0.090 (0.029) *0.046*	0.076 (0.026) *0.028*	
A3α5α-C (Androsterone-C)	5.041 (1.038)** *3.510*	2.876 (0.983) *1.405*	0.01	5.703 (1.294)** *4.529*	1.156 (0.238) *0.926*	<0.01
A3α5β (Etiocholanolone)	0.049 (0.012) *0.027*	0.074 (0.019) *0.039*		0.033 (0.007) *0.021*	0.025 (0.006) *0.016*	
A3α5β-C (Etiocholanolone-C)	1.098 (0.287) *0.526*	1.217 (0.792) *0.142*		0.892 (0.382) *0.354*	0.407 (0.135) *0.196*	
A3β5α (Epiandrosterone)	0.068 (0.008) *0.059*	0.143 (0.049) *0.086*		0.196 (0.106) *0.092*	0.190 (0.123) *0.066*	
A3β5α-C (Epiandrosterone-C)	2.461 (0.604) *1.408*	1.762 (1.146) *0.441*		21.91 (18.32)* *4.107* ^*#*^	1.992 (0.456) *1.109*	<0.05
AT-7α (5-Androstene-3b,7α,17b-triol)	0.397 (0.045) *0.348*	0.289 (0.049) *0.240*		0.384 (0.077) *0.295*	0.233 (0.027) *0.226*	
AT-7 β (5-Androstene-3 β,7 β,17β-triol	0.271 (0.025) *0.227*	0.207 (0.032) *0.187*		0.291 (0.081) *0.198*	0.185 (0.020) *0.183*	

Mean steroid concentrations (nM) ± (SEM). Italics—median concentrations (nM)

Analysis: three-way ANOVA—significant effects and interactions of Pregnenolone: illness [*F*(1,140) = 25.02, *p* < 0.01], sex [*F*(1,140) = 11.40, *p* < 0.01], and age group × illness interaction [*F*(1,140) = 20.91, *p* < 0.01], other effects were not significant (*p* > 0.05). Pregnenolone-C: illness [*F*(1,138) = 20.62, *p* < 0.01], sex [*F*(1,138) = 18.33, *p* < 0.01], age group × sex interaction [*F*(1,138) = 6.48, *p* = 0.01]. 20-alpha dihydropregnenolone: illness [*F*(1,133) = 33.63, *p* < 0.01], sex [*F*(1,133) = 69.78, *p* < 0.01], age group × illness interaction [*F*(1,133) = 11.68, *p* < 0.01]. 20-alpha dihydropregnenolone-C: illness [*F*(1,113) = 21.00, *p* < 0.01]. Allopregnanolone: illness [*F*(1,131) = 46.08, *p* < 0.01], sex [*F*(1,131) = 63.47, *p* < 0.01], age group [*F*(1,131) = 18.36, *p* < 0.01], age group × sex interaction [*F*(1,131) = 5.29, *p* = 0.02], age group × illness interaction [*F*(1,131) = 33.30, *p* < 0.01], age group × sex × illness triple interaction [*F*(1,131) = 14.38, *p* < 0.01]. Isopregnanolone-C: sex × illness interaction [*F*(1,104) = 4.4, *p* ≤ 0.04], age group × sex × illness triple interaction [*F*(1,104) = 5.1, *p* = 0.03]. Epipregnanolone-C: sex [*F*(1,139) = 100.8, *p* < 0.01], age group × sex × illness triple interaction [*F*(1,139) = 12.8, *p* < 0.01]. DHEA: illness [*F*(1,140) = 43.33, *p* < 0.01] and age group × illness interaction [*F*(1,140) = 14.78, *p* < 0.01]. DHEA-C: illness [*F*(1,139) = 36.74, *p* < 0.01], sex [*F*(1,139) = 10.43, *p* < 0.01], age group [*F*(1,139) = 21.43, *p* < 0.01]. DHEA-7o: illness [*F*(1,114) = 7.67, *p* < 0.01] and sex × illness interaction [*F*(1,114) = 5.04, *p* = 0.03]. Androstenediol: illness [*F*(1,140) = 45.37, *p* < 0.01], sex [*F*(1,140) = 4.23, *p* = 0.04], age group × illness interaction [*F*(1,140) = 7.46, *p* < 0.01]. Androsterone-C: sex [*F*(1,139) = 7.15, *p* < 0.01] and age group × illness interaction [*F*(1,139) = 4.89, *p* = 0.03]. Etiocholanolone: illness [*F*(1,111) = 5.4, *p* = 0.02] and age group × illness interaction [*F*(1,111) = 8.4, *p* < 0.01]. Etiocholanolone-C: illness [*F*(1,138) = 5.3, *p* = 0.02]. Epiandrosterone: illness [*F*(1,113) = 467.29, *p* < 0.01], sex [*F*(1,113) = 8.01, *p* < 0.01], sex × illness interaction [*F*(1,113) = 7.22, *p* < 0.01], age group × illness interaction [*F*(1,113) = 9.36, *p* < 0.01]. Epiandrosterone-C: illness [*F*(1,139) = 35.41, *p* < 0.01], sex [*F*(1,139) = 25.42, *p* < 0.01], age group [*F*(1,139) = 35.41, *p* < 0.01], age group × sex interaction [*F*(1,139) = 6.33, *p* = 0.01], age group × illness interaction [*F*(1,139) = 8.76, *p* < 0.01], age group × sex × illness triple interaction [*F*(1,139) = 6.87, *p* < 0.01]. 5-Androstene-3β,7α,17β-triol (AT-7alpha): illness [*F*(1,139) = 32.78, *p* < 0.01] and sex × illness interaction [*F*(1,139) = 5.6, *p* = 0.02]. 5-Androstene-3β,7β,17β-triol (AT-7beta): illness [*F*(1,140) = 26,25, *p* < 0.01]. Cortisol, allopregnanolone-C, androstenedione and androsterone: no significant effects

AF I and AF II (autistic females group I and II), CF I and CF II (control females group I and II)

* p < 0.05; ** p < 0.01 significant differences in steroid concentrations between autistic and control children within age groups

^#^ *p* < 0.05; ^##^ *p* < 0.01 significant differences between older and younger children, either autistic or control. “C” after steroid name denotes conjugate form

Markedly higher levels of many steroid hormones were found in the saliva of male and female autistic children than in healthy controls. The effects were more pronounced in older children, particularly in boys. In younger autistic boys (AM I), post hoc analysis showed significantly higher levels of only 4 steroids (DHEA-C, androsterone-C, 5-androstene-3β,7α,17β-triol, 5-androstene-3β,7β,17β-triol) as compared to controls (CM I). However, in older autistic boys (AM II), the levels of 17 steroids were markedly higher than in the controls (CM II), (Table 3): 6 of them were C21 steroids (pregnenolone, 20-alpha dihydropregnenolone, 20-alpha dihydropregnenolone-C, allopregnanolone, isopregnanolone-C, and epipregnanolone-C) and 11 were C19 steroids (DHEA, DHEA-C, DHEA-7o, androstenediol, androsterone-C, etiocholanolone, etiocholanolone-C, epiandrosterone, epiandrosterone-C, 5-androstene-3β,7α,17β-triol, 5-androstene-3β,7β,17β-triol). There were no significant differences in cortisol levels between the groups of autistic and control boys.

Elevated concentrations of salivary steroids were also observed in autistic girls (Table 4). In younger girls (AF I), the levels of two androgenic C19 steroids (DHEA-C and androsterone-C) were significantly higher than in controls (CF I), but in older autistic girls (AF II), 4 steroids from the C21 group (pregnenolone, pregnenolone-C, 20-alpha dihydropregnenolone, allopregnanolone) and 4 from the C19 group (DHEA, androstenediol, androsterone-C, epiandrosterone-C) were significantly higher than in controls (CF II). As in boys, the levels of cortisol did not differ between autistic and healthy girls. The concentrations of two steroids, DHEA-C and androsterone-C, were universally increased in all groups of autistic children.

**Fig. 1:**
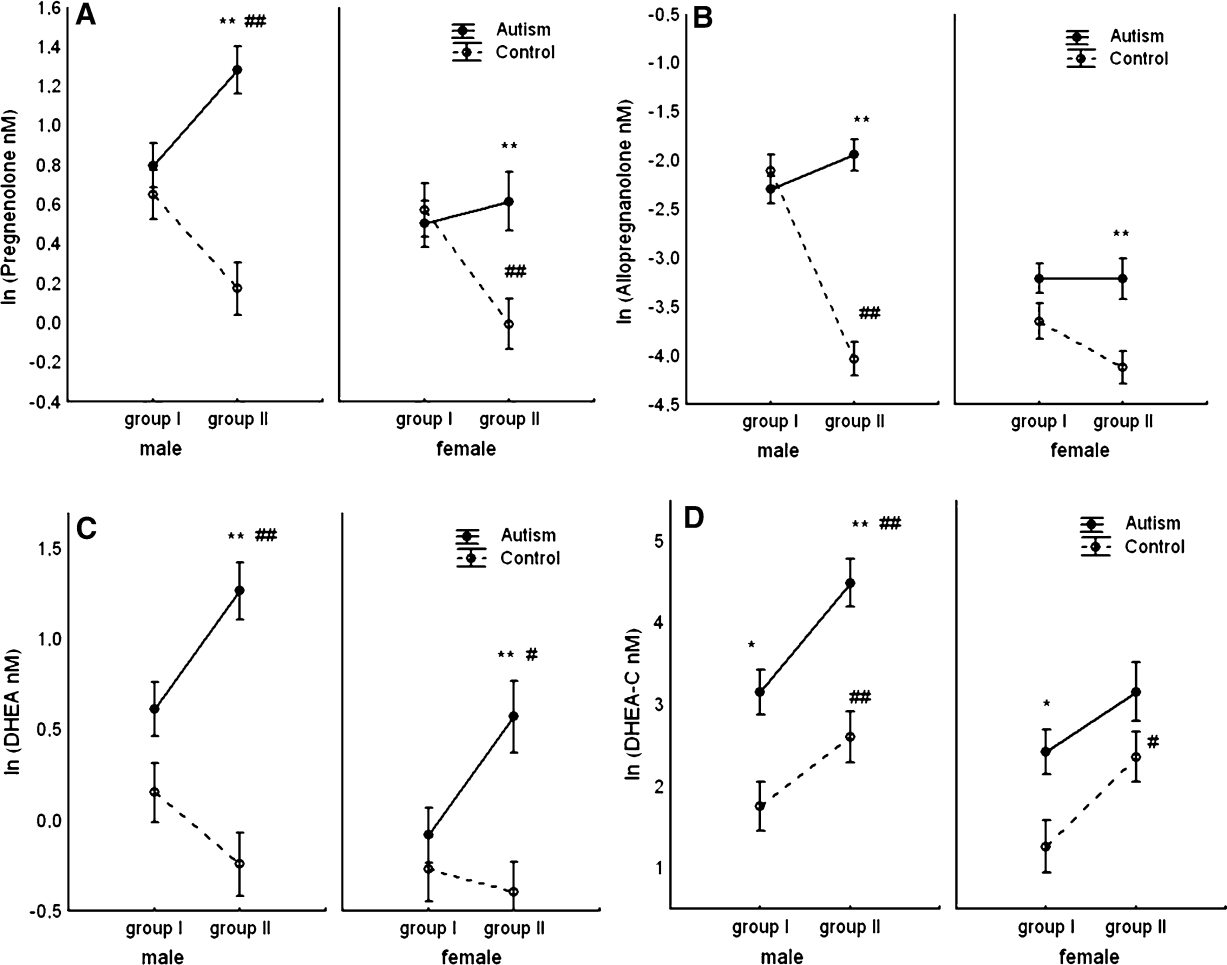
Distinct patterns of developmental changes in salivary levels of neuroactive steroids (pregnenolone, allopregnanolone, DHEA and DHEA-C) in autistic children than in healthy controls. Groups I and II refer to experimental age groups. Data represent natural logarithms of mean (nM) concentrations. Significant differences between autistic and control groups: *p < 0.05 and **p < 0.01. Significant differences between older and younger groups of either autistic or control children: #*p* < 0.05, ##*p* < 0.01

The results also revealed divergences in the dynamics of developmental changes of steroids between autistic and healthy children. While in autistic boys, the levels of several steroids (pregnenolone, 20-alpha-dihydropregnenolone, DHEA, epiandrosterone, epiandrosterone-C, allopregnanolone) increased during the 4 years of development separating the older (AM II) from the younger (AM I) group, in control boys the concentrations of these steroids somewhat declined during this period. This resulted in greater differences in the levels of many steroids between the groups of older autistic and healthy children than between equivalent younger groups. The concentrations of DHEA-C increased roughly in parallel during the four developmental years in autistic and healthy children, achieving much higher levels in autistic groups. Data shown in Fig. 1 illustrate distinctions in the developmental changes of four steroids/neurosteroids (pregnenolone, allopregnanolone, DHEA and DHEA-C) between autistic and control children.

**Fig. 2:**
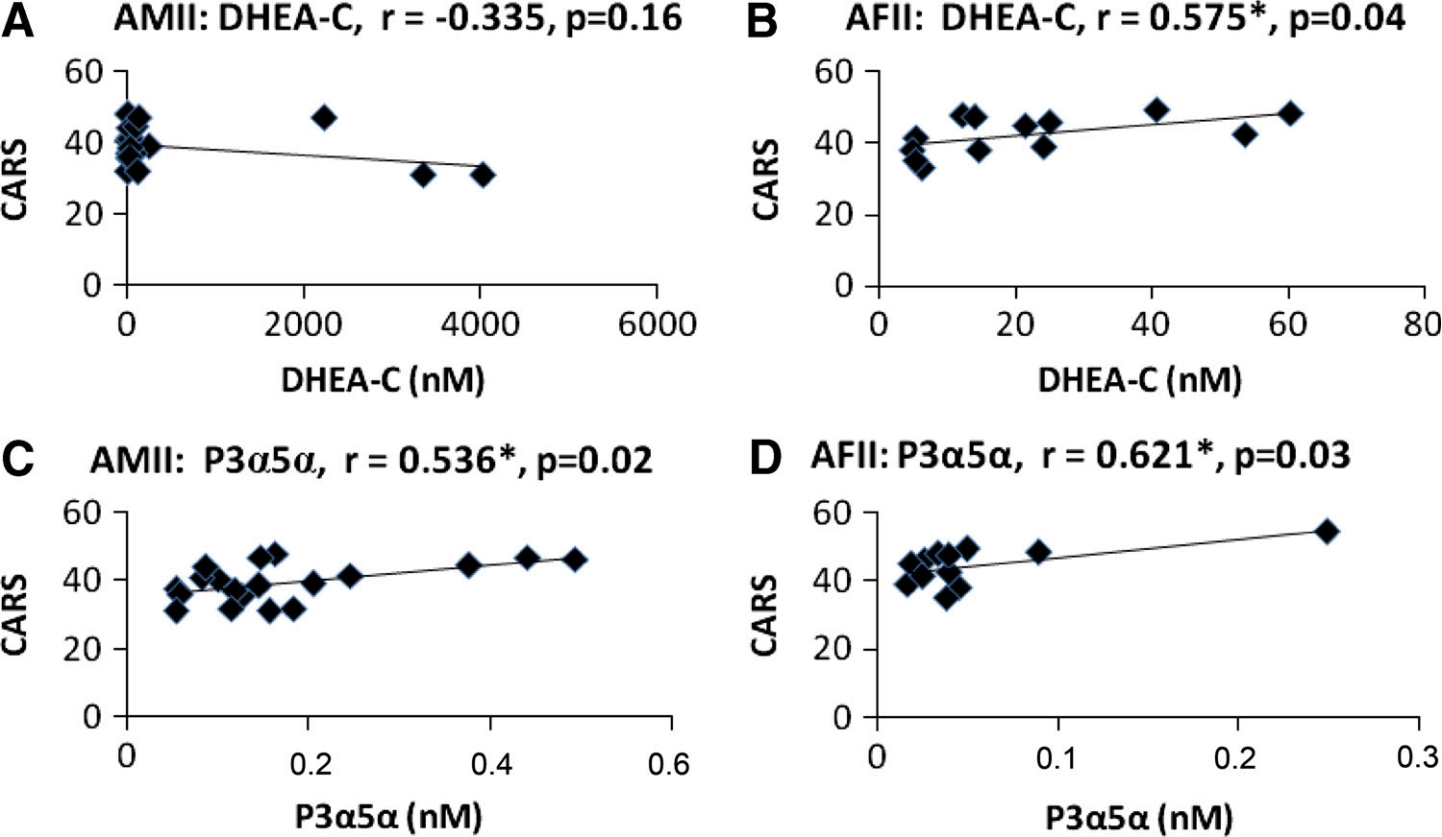
Scatter plots of correlations between salivary levels of two major neurosteroids (DHEA-C and allopregnanolone/P3α5α) and CARS scores in older autistic boys and girls. **a**, **c** Autistic boys (AMII); **b**, **d** autistic girls (AFII). *Stars* denote statistically significant correlations; *p* < 0.05

We attempted to correlate the steroid levels with autism severity. In older autistic girls (AF II) there were moderate to strong positive correlations between the CARS scores and levels of several steroids: pregnenolone (*r* = 0.70, *p* = 0.007), pregnenolone-C (*r* = 0.66, *p* < 0.014), 20-alpha dihydropregnenolone (*r* = 0.752, *p* = 0.003), allopregnanolone (*r* = 0.621, *p* = 0.031), allopregnanolone-C (*r* = 0.583, *p* = 0.036), DHEA (*r* = 0.618, *p* = 0.024), DHEA-C (*r* = 0.575, *p* < 0.04), DHEA-7o (*r* = 0.608, *p* = 0.027), androstenediol (*r* = 608, *p* = 0.027), and 5-androstene-3β,7α,17β-triol (*r* = 0.707, *p* = 0.007). Moderate positive correlations between CARS scores and concentrations of allopregnanolone (*r* = 0.536, *p* = 0.02) and isopregnanolone-C (*r* = 0.422, *p* = 0.072) were likewise found in older autistic boys (AMII). Interestingly, in the latter group there were also weak negative correlations of CARS scores with the levels of some steroids: DHEA (*r* = −0.314, *p* = 0.177), DHEA-C (*r* = −0.335, *p* = 0.161), and epiandrosterone-C (*r* = −0.291, *p* = 0.29). Negative correlations between CARS scores and the concentrations of androsterone (*r* = −0.497, *p* = 0.022), DHEA-7o (*r* = −0.488, *p* = 0.021), and DHEA (*r* = −0.283, *p* = 0.2) were likewise found in younger autistic girls (AF I). For younger autistic boys such correlations were weak and mostly negative (for DHEA-C, *r* = −0.225, *p* = 0.50). A few autistic children had unusually high levels of some steroids. One boy from the AM I group, who had the highest DHEA and DHEA-C levels in his group, had the total CARS score 37.5, which was below the average for his group, and two boys from the AM II group, who had the highest DHEA and DHEA-C levels in their group, had the lowest CARS scores in their group (31.0). On the other hand, a girl from the AF II group with uppermost levels of DHEA, DHEA-C and several other steroids, had the second highest CARS score of all autistic children (54.5). Scatter plots of correlations between CARS scores and salivary levels of two major neurosteroids (DHEA-C and allopregnanolone/P3α5α) in older autistic boys and girls are shown in Fig. 2.

## Discussion

To the best of our knowledge, this is the first study documenting the association of autism with significantly raised levels of many steroid hormones (both C21 and C19) in the saliva of prepubertal autistic children of both sexes, as compared to healthy controls. The effect was more pronounced in older children, particularly in boys. While in older autistic male and female children the concentrations of many steroids, especially androgens, were markedly elevated, in younger autistic girls and boys the levels of only 2–4 steroids were increased. The levels of DHEA-C and androsterone-C were universally higher in all groups of autistic children than in the controls, which suggest that these steroids may potentially serve as autism biomarkers for all prepubertal children.

Saliva has progressively become a preferred specimen choice for steroid analysis for some groups of patients, because of its convenient, noninvasive and stress-free collection [31]. This is especially important for autistic children, who often have exaggerated stress reactions (which alter the profile of secreted steroid hormones). Levels of steroids in saliva generally reflect their blood levels and are several-fold lower than those in serum [32, 33], although concentration ratios for different steroids in these body fluids depend on steroid polarity. Unconjugated, lipid-soluble steroids (e.g. cortisol, pregnenolone, androgens) easily pass through capillary walls and their salivary concentrations have been assessed to represent about 10 % of serum levels, but the levels of conjugated steroids may represent only about 1–2 % of those in serum [31–33]. At present, we have no data comparing the levels of all analyzed steroids in saliva and serum (such a study is clearly needed). However, comparing the concentrations of selected salivary steroids in 8-year-old healthy boys from our study with those in serum measured by others [34] gave the saliva to serum ratios of about 1.5 % for DHEA-C/DHEA-S and about 15 % for DHEA—the values analogous to the estimates of Vining [33]. The above factors have to be taken into consideration, when comparing steroid concentrations in saliva and blood, but they do not diminish the practical value of comparing salivary steroid levels between different groups of patients, or its utility in tracking hormonal changes in patients during the course of therapy.

The most prominent steroids in children’s saliva were the polar conjugates of DHEA, pregnenolone, androsterone, epiandrosterone, and 20α-dihydropregnenolone. We did not separate these fractions into different species; but it is known that in human serum the polar conjugates of pregnenolone and DHEA consist mostly of sulfate forms (pregnenolone sulfate/PS and DHEAS), although conjugate fractions of 5α-reduced androgens (androsterone, epiandrosterone) may also contain substantial portions of glucuronides [35]. Likewise, polar conjugates of reduced progesterone metabolites (allo-, iso-, and epipregnanolones) may contain sulfates with an admixture of glucuronides.

The most likely source of amplified steroid synthesis in autistic children seems to be the adrenal cortex, because in humans this tissue is the main source of DHEA and DHEAS, whose levels were most highly elevated. Furthermore, all study participants were prepubertal. Nevertheless, some involvement of steroidogenic tissues of other organs such as the gonads, liver, stomach, duodenum, lungs, spleen, kidneys, skin, and salivary glands cannot be ruled out. DHEA and DHEAS are weak androgens that undergo striking developmental changes in humans: in fetuses and newborns they are produced in large quantities by the fetal zone of the adrenal cortex, which in the first postnatal months undergoes apoptotic involution, and morphological and functional reorganization [36]. As a result, in the first years of life the adrenals secrete very small quantities of androgens. Prior to gonadal maturation, during adrenarche (at 6–9 years old) the adrenal cortex is transformed and the reticular zone (RZ) emerges, which synthesizes DHEA, DHEAS and other androgens [37]. The blood levels of DHEA and DHEAS peak at years 20–25 of life and then decline with aging. Because the amounts of synthesized androgens correlate with the size of adrenals [38], this suggests that amplified steroid release in prepubertal autistic children—indicative of precocious adrenarche [39]—may be due to early maturation, hyperplasia or hypertrophy of the adrenal RZ. We have not analyzed ACTH levels in this study; but lack of significant differences in cortisol concentrations between experimental groups suggests that the RZ (but not zona fasciculata) primarily contributes to augmented steroid secretion in autism. This pattern is consistent with accelerated adrenarche, typified by a rise in the release of adrenal androgens, but not glucocorticoids. However, we cannot exclude the possibility of altered diurnal cortisol rhythms in autism. Lakshmi Priya et al. [40] observed such disturbances along with raised cortisol levels in autistic Indian children, but their patient population was demographically more diverse than ours; primarily their most affected low functioning group was undernourished, which could have contributed to augmented cortisol secretion (our patients did not have this problem).

Our results are consistent with the findings of Geier and Geier [10] and El-Baz et al. [41], who reported hyperandrogenemia in American and Egyptian autistic children, as well as with the observations of Ruta et al. [11] in adults with autism spectrum conditions. They are, however, at variance with the report of Tordjman et al. [14], who did not find elevated levels of DHEA in the serum of most autistic children, except for those with aggression [15], and they differ from the results of Strous et al. [12], who found diminished plasma levels of DHEAS in adults with autism. What could cause such discrepancies? Reduced DHEAS secretion in autistic adults could possibly be explained by adrenal exhaustion following earlier overactivity, but divergent results in children require a different explanation. Theoretically, they could be due to genetic heterogeneity of the studied populations. Abnormal metabolism of cholesterol has been found in nearly 20 % of autistic patients [18] and autism typically accompanies Smith–Lemli–Opitz syndrome (SLOS)—a genetic disorder of impaired cholesterol synthesis [42]. Yet, SLOS cases represent only a small percent of autistic cases and SLOS prevalence in Caucasians (1/20,000–1/70,000) is several hundred times lower than the current prevalence of autism. In our original group of autistic patients only one boy had a syndactyly characteristic of SLOS and this child was excluded from the present study. Autistic traits have also been linked with the gene *CYP11B1* (cytochrome P45011B1), which is related to adrenal steroid metabolism [43]. While its mutations may lead to adrenal hyperplasia and androgen excess, most individuals with such defects do not suffer from autism. Excessive androgen secretion may also ensue from congenital adrenal hyperplasia resulting from a deficiency of 21-hydroxylase, a milder version of which may occur in about 1 % of Caucasians [44]. We have not measured the enzymes of sex hormone pathways, nor genetic defects in steroid metabolism, hence we cannot exclude such anomalies in some patients. Nevertheless, it has to be emphasized that—with an exception of SLOS—autism is not generally characteristic of individuals having the above conditions. More likely, increased steroid levels are markers of complex autism pathology.

Another factor potentially responsible for the disparities in steroid metabolism between different autistic populations could be the diverse environmental or iatrogenic exposure to hormone disruptors such as heavy metals and other toxins. For example, mercury, implicated in autism pathogenesis by a number of studies [rev. 4, 45] was shown to disturb adrenal steroidogenesis by increasing mitochondrial concentration of heme and cytochrome P-450, resulting in elevation of cholesterol side chain cleavage and augmented synthesis of DHEA [46]. Such an effect may contribute to premature puberty in children exposed to vaccines containing thimerosal [47]—an organomercurial which has been added to pediatric vaccines for decades.

Excessive steroid secretion, regardless of its causes, in prepubertal autistic children points to precocious adrenarche and may be predictive of early puberty. The anomalous steroid milieu during childhood may disturb brain development and functioning, as steroids easily penetrate the blood brain barrier and many are neuroactive. Pregnenolone-S and DHEAS are allosteric antagonists of GABAA receptors [22, 28] and agonists of NMDA receptors [23]; hence their excessive levels in the brain may synergistically enhance neuronal excitation. Augmented levels of allopregnanolone (also termed 3α,5α-tetrahydroprogesterone) and androsterone, which are allosteric agonists of GABAA receptors and are neuroinhibitory [22, 27], may counteract the stimulant actions of pregnenolone-S and DHEAS, so the final neurophysiological outcome may depend on the relative ratios of stimulatory to inhibitory steroids in a person. DHEA-C attained particularly high levels in the saliva of autistic children (its median concentrations reached 237, 445, 270, and 194 % of control values in groups AM I, AM II, AF I and AF II, respectively). Such excess of an excitatory steroid may amplify the neurostimulant effects resulting from reported deficits in GABA neurotransmission [48] and augmented activity of glutamate [49] in persons with autism, contributing to increased anxiety, sleep disturbances and seizures, which are often comorbid with ASDs [6].

Raised levels of androsterone-C in autistic children may also be clinically relevant, as androsterone sulfate (constituting a major fraction of androsterone-C) in addition to being a precursor of neuroinhibitory androsterone, itself possesses opioid-like features, inducing agitation, hypermotility, EEG disturbances and stereotypy [50]. Such effects may be partly responsible for hypoalgesia, hyperactivity, as well as stereotypical and self-injurious behaviors in autistic children, whereas amplified secretion of androstenediol and its metabolite, testosterone, may promote impulsivity, anger and aggression. Augmented production of 7α-androstenetriol, which possesses immune stimulating and antiglucocorticoid features [51], may promote inflammatory processes, autoimmunity and other immune dysfunctions that are often comorbid with autism [52, 53]. Without a doubt, excessive secretion of neuroactive steroids may disrupt brain development and functioning by interacting directly and indirectly with neurotransmitter receptors (GABA, glutamate, opioids) and the immune system, contributing to a range of clinical and behavioral manifestations of autism.

Steroids have a remarkable diversity of physiological functions and may play multifaceted roles in autism. Some, while bolstering autism symptoms, may also be neuroprotective. For example, we have recently shown that DHEAS prevents thimerosal-induced accumulation of extracellular glutamate in the rat brain [54]. As excessive glutamate activity (also found in autism) is linked with excitotoxicity and neuronal death, enhanced synthesis of DHEAS in autistic children may partially protect their brains against the neurotoxic effects of glutamate and mercurials, as it protects against other types of CNS injuries [55, 56]. Sulfosteroids may also shield the body and brain from the toxicity of mercurials by facilitating their elimination [57]. From this perspective, amplified steroid synthesis in autism may be viewed as mobilization of the body’s defense mechanisms against metallic (and potentially other) toxins. Accelerated adrenarche and production of large quantities of sulfated steroids may partly explain our earlier observation of seemingly more efficient mercury elimination by older autistic children than by younger ones [26]. On the other hand, hyperandrogenism may potentiate autistic traits, leading to behavioral anomalies such as aggression against self and the others. For such patients, treatment with antiandrogenic medications to normalize androgen levels has been proposed [58]. The long-term effects of such therapy in autistic children should be evaluated.

In our study, the correlations of steroid levels with autism severity gave equivocal results, depending on the sex and age of the child, and on steroid type. For example, in older autistic girls, higher concentrations of most steroids correlated positively with CARS scores, but in older boys—while the levels of allopregnanolone and isopregnanolone also correlated positively with CARS—the levels of DHEA, DHEA-C and epiandrosterone correlated negatively. In younger girls and boys, higher levels of many steroids also correlated mostly negatively with autism severity. This suggests that augmented secretion of certain steroids may be protective for some autistic children. Such intricate relationships, which need to be confirmed in larger cohorts of children, reflect the multifaceted physiological and neurochemical functions of steroid hormones.

The specific environmental, iatrogenic, genetic, and epigenetic factors responsible for aberrant steroid metabolism in autism remain to be elucidated. Involvement of epigenetic mechanisms—linking genes with the environment—is suggested by studies conducted on lymphoblasts from siblings discordant for autism diagnosis, which showed increased expression of genes associated with steroid biosynthesis along with genes involved in brain development in autism [19]. Augmented levels of certain steroids may in turn alter the expression of genes implicated in autism pathogenesis such as Reelin and RORA [59, 60].

While comparing salivary levels of steroid hormones in autistic and healthy children was the primary focus of the present study, it also provided additional relevant observations. (1) Generally, there was no significant difference between autistic and control children in demographics, or maternal and paternal age at child birth. (2) Three of the four groups of autistic children did not differ significantly from healthy groups in birth-linked parameters such as weight, head circumference, gestational age, or Apgar score. These findings, which were discussed in detail in our earlier publication (26), seem to suggest that (at least in our study population) broadly defined postnatal environmental factors are the predominant contributors to autism etiology.

## Conclusion

Augmented salivary levels of many steroid hormones have been found in prepubertal autistic children, suggestive of precocious adrenarche and predictive of early puberty. Several of these steroids are known to affect the functioning of the nervous and immune systems. While excessive secretion of some steroids (e.g. androgens) may adversely influence child development and behavior, contributing to autistic symptoms, it may also play a neuroprotective role and represent the body’s defense reaction to heavy metal intoxication.

### Study limitations and perspectives

The present findings do not allow assessment of the exact role of anomalous steroid synthesis in autism pathogenesis, nor do they permit prediction if limiting production of certain steroids would be clinically beneficial or harmful for autistic children. However, they open an avenue for further research and prospective therapies. (1) It remains to be established if raised steroid levels are universal autism biomarkers, or are characteristic only of some autistic populations. (2) The contributions of biological/genetic and environmental/iatrogenic factors in augmented steroid biosynthesis in autism should be examined. (3) The potential pathogenic and/or protective functions of different steroids in autism need to be clarified. (4) Our data were derived from relatively small groups of patients recruited by a single medical center and should be replicated in a larger multicenter study. International studies would help to address a potential involvement of environmental or iatrogenic factors in alterations of steroid synthesis in autism.

## Electronic supplementary material

Below is the link to the electronic supplementary material.

Supplementary material 1 (DOCX 19 kb)
