# Supplementary material for: Marked elevation of adrenal steroids, especially androgens, in saliva of prepubertal autistic children
Source: Eur Child Adolesc Psychiatry. 2013 Sep 17;23(6):485–98. doi: 10.1007/s00787-013-0472-0 (PMC4042015; doi:10.1007/s00787-013-0472-0)
Supplement: Supplementary file 1 — Supplementary material 1 (DOCX 19 kb) [file 787_2013_472_MOESM1_ESM.docx]

Supplementary material:

| Test of the difference between steroid concentrations in immediately processed 20 replicates of pooled samples and the same number of replicates after two steps of freezing | | | | | | | | | | |
| --- | --- | --- | --- | --- | --- | --- | --- | --- | --- | --- |
| **Steroid** | **Unfrozen** | | |  | **Frozen** | | |  | **Mann-Whitney test** (p-value) | **Mann-Whitney test with correction for multiplicity** |
|  | Median | 95.0% LCL | 95.0% UCL |  | Median | 95.0% LCL | 95.0% UCL |  |  |  |
| Pregnenolone | **4.257** | 4.079 | 4.748 |  | **4.613** | 4.11 | 4.893 |  | 0.424883 | NS |
| Conjugated pregnenolone | **4.073** | 3.487 | 4.324 |  | **3.819** | 3.383 | 4.195 |  | 0.440750 | NS |
| 20α-Dihydropregnenolone | **1.304** | 1.221 | 1.368 |  | **1.162** | 1.108 | 1.244 |  | 0.004703 | NS |
| Conjugated 20α-dihydropregnenolone | **2.173** | 1.951 | 3.058 |  | **2.204** | 1.988 | 2.43 |  | 0.694891 | NS |
| Dehydroepiandrosterone | **2.428** | 2.278 | 2.549 |  | **2.525** | 2.345 | 2.621 |  | 0.285305 | NS |
| Conjugated dehydroepiandrosterone | **9.751** | 8.691 | 11.04 |  | **9.371** | 8.638 | 10.13 |  | 0.297677 | NS |
| Androstenediol | **1.682** | 1.657 | 1.791 |  | **1.581** | 1.535 | 1.614 |  | 0.007113 | NS |
| Conjugated androstenediol | **11.59** | 10.38 | 12.48 |  | **10.97** | 10.11 | 11.77 |  | 0.350702 | NS |
| Androstenedione | **0.396** | 0.353 | 0.436 |  | **0.389** | 0.347 | 0.438 |  | 0.818149 | NS |
| Androsterone | **0.078** | 0.067 | 0.086 |  | **0.072** | 0.057 | 0.085 |  | 0.323482 | NS |
| Conjugated androsterone | **1.366** | 1.21 | 1.468 |  | **1.417** | 1.33 | 1.535 |  | 0.440750 | NS |
| Etiocholanolone | **0.05** | 0.043 | 0.053 |  | **0.044** | 0.037 | 0.055 |  | 0.542772 | NS |
| Conjugated etiocholanolone | **0.127** | 0.06 | 0.258 |  | **0.163** | 0.109 | 0.205 |  | 0.350702 | NS |
| Epiandrosterone | **0.199** | 0.192 | 0.208 |  | **0.188** | 0.179 | 0.204 |  | 0.379332 | NS |
| Conjugated epiandrosterone | **0.86** | 0.754 | 1.27 |  | **1.15** | 1.014 | 1.325 |  | 0.101729 | NS |
| 5-Androstene-3β,7α,17β-triol | **0.332** | 0.305 | 0.382 |  | **0.307** | 0.252 | 0.354 |  | 0.228694 | NS |
| 5-Androstene-3β,7β,17β-triol | **0.357** | 0.313 | 0.387 |  | **0.332** | 0.304 | 0.368 |  | 0.155570 | NS |
| Conjugated epipregnanolone | **0.199** | 0.12 | 0.234 |  | **0.212** | 0.132 | 0.446 |  | 0.409356 | NS |
| Allopregnanolone | **0.049** | 0.04 | 0.056 |  | **0.045** | 0.035 | 0.052 |  | 0.218406 | NS |
| Conjugated allopregnanolone | **0.082** | 0.068 | 0.115 |  | **0.132** | 0.078 | 0.162 |  | 0.076431 | NS |
| Conjugated isopregnanolone | **0.702** | 0.59 | 0.755 |  | **0.692** | 0.61 | 0.882 |  | 0.524987 | NS |
| 7-oxo-Dehydroepiandrosterone | **1.865** | 1.754 | 2.01 |  | **1.871** | 1.809 | 1.994 |  | 0.776391 | NS |
| *NS=not significant (p>0.05),LCL=lower confidence limit, UCL=upper confidence limit* | | | | | | | |  |  |  |
